# Supplementary material for: Causal attributions shape the formation of novel ability self-beliefs
Source: Commun Psychol. 2026 Jun 5;4:90. doi: 10.1038/s44271-026-00479-8 (PMC13237141; doi:10.1038/s44271-026-00479-8)
Supplement: Supplementary file 2 — Supplementary Material [file 44271_2026_479_MOESM2_ESM.pdf]

Supplementary Materials for:

Causal attributions shape the formation of novel ability self-beliefs

Annalina V Mayer, Alexander Schröder, David S Stolz, Nora Czekalla, Frieder M Paulus, Sören Krach, Tobias Kube, Laura Müller-Pinzler

Contents

Supplementary Figures **2**
- Figure S1: Percentage of retrospectively perceived agent interference 2
- Figure S2: Bayesian Model Selection 3
- Figure S3: Simulated data demonstrating how the attribution weight factor of the winning model affects performance expectation trajectories 4

Supplementary Tables **5**
- Table S1: PSIS-LOO scores 5
- Table S2: Posterior predictive check: linear mixed model on simulated performance expectations 6
- Table S3: Correlations of model parameters (winning computational model M5) 7

Supplementary Notes **8**
- Supplementary Note 1: Participant instruction 8
- Supplementary Note 2: Sample size determination and post-hoc sensitivity analysis 9

References **10**

Supplementary Figures

Figure S1: Percentage of retrospectively perceived agent interference.

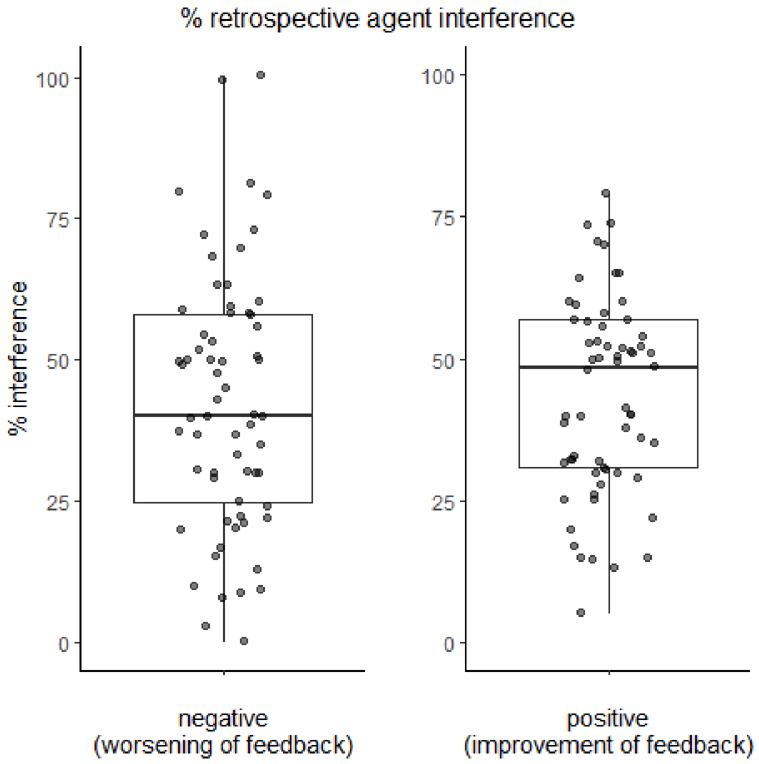

Note. Apart from trial-by-trial attributions of feedback in the Agent condition, we also measured participants' belief in agent interference in a follow-up survey after completion of the LOOP task. Specifically, participants were asked to retrospectively rate the frequency of agent interference: "Think about the trials in which it was possible for the feedback to be manipulated in a positive direction, i.e., improved. Looking back, in what percentage of cases do you think the computer manipulated the feedback?" and "Think about the trials in which it was possible for the feedback to be manipulated negatively, i.e., worsened. Looking back, in what percentage of cases do you think the computer manipulated the feedback?"

Figure S2: Bayesian Model Selection.

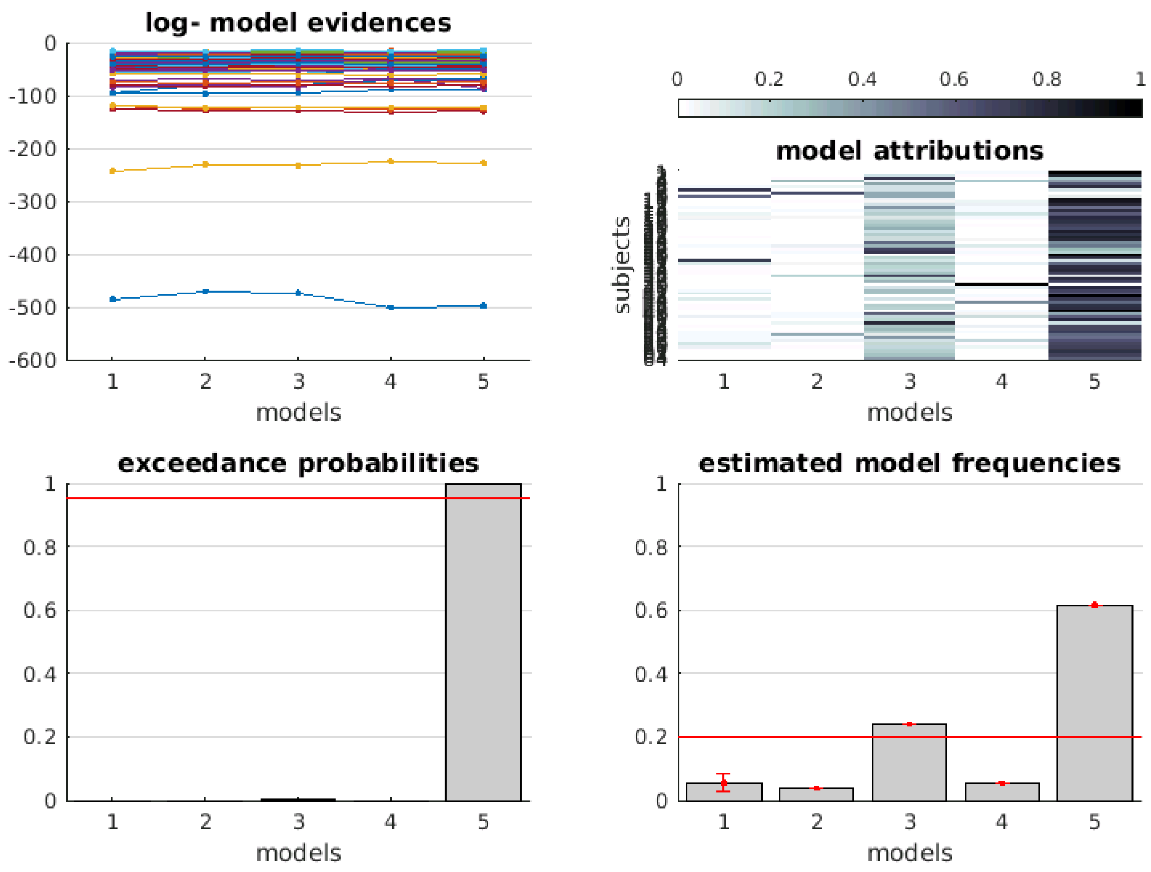

Note. Model 5 (Attribution Shrink Model) emerged as the winning model with a protected exceedance probability pxp = .999. and an estimated model frequency of 61.49.

Figure S3: Simulated data demonstrating how the attribution weight factor of the winning model affects performance expectation trajectories.

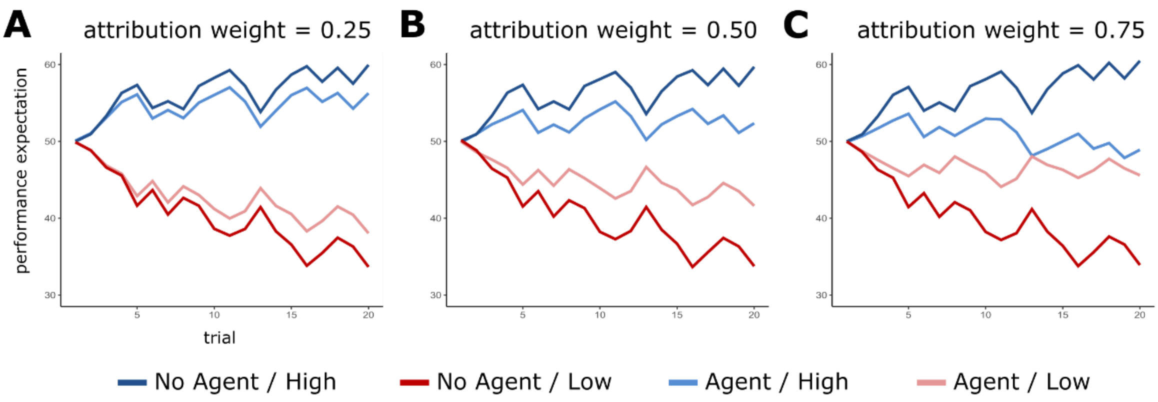

Note. These plots show how the attribution weight parameter in the winning model attenuates trial-by-trial learning in the Agent condition when feedback is attributed externally. It is important to note that this data was simulated based on the assumptions of an “optimal learner” in regard to this task. This means that expectations started at 50 in all conditions, the self-beliefs were updated according to the feedback, no counterfactual updates (e.g., a positive update following a negative prediction error) were considered, and only a small degree of noise was fitted onto the expectation ratings. The simulation assumed a learning rate for positive prediction errors of 0.15 and one for negative prediction errors of 0.2. A feedback weighting factor *w* of 0.9 was used. In the Agent condition, 40% of all trials were considered to be manipulated by the agent. **A** shows data with an attribution weight factor of 0.25, **B** of 0.5, and **C** of 0.75.

Supplementary Tables

Table S1: PSIS-LOO scores.

Model	PSIS LOO	LOO SE	PSIS LOO Diff	LOO SE Diff	percent. $\hat{k}$ > 0.7	No. est. param.
Model 1	-3366.86	525.62	-122.55	-8.12	0.76	7
Model 2	-3317.81	509.88	-73.5	-23.86	1.29	9
Model 3	-3268.48	515.33	-24.17	-18.41	0.92	8
Model 4	-3287.03	532.94	-42.72	-0.8	1.11	9
Model 5	-3244.31	533.74	-	-	0.92	8

Note. PSIS-LOO = sum score of approximate leave-one-out cross-validation (LOO) using Pareto-smoothed importance sampling (PSIS); LOO-SE = standard error of PSIS-LOO; LOO-Diff = difference in expected predictive accuracy for all models in reference to the winning model with the highest PSIS-LOO (M5, Attribution Shrink Model; highlighted row) and standard error of these differences; percentage of $\hat{k}$ (estimated shape parameters of the generalized Pareto distribution) that exceed 0.7 (Vehtari et al. 2016); No. est. param. = number of parameters the model estimates (learning rates, initial expectation ratings for each condition, weight, and shrink factors where applicable).

Table S2: Posterior predictive check: linear mixed model on simulated performance expectations

Fixed effects

	Beta	SE	95% CI lower	95% CI upper	*t*	*p*
Intercept	40.303	1.899	36.580	44.027	21.221	< 0.001
Trial	-0.228	0.078	-0.381	-0.075	-2.926	0.003
Interference	-1.106	0.239	-1.575	-0.637	-4.621	< 0.001
Ability	6.535	0.239	6.066	7.004	27.304	< 0.001
Trial * Interference	0.041	0.042	-0.040	0.122	0.986	0.324
Trial * Ability	0.480	0.042	0.399	0.562	11.568	< 0.001
Interference * Ability	-8.538	0.479	-9.477	-7.600	-17.838	< 0.001
Trial * Interference * Ability	-0.466	0.083	-0.629	-0.303	-5.611	< 0.001

Random effects

	SD	Correlation
Participant (Intercept)	12.13	
Participant (Trial)	0.60	
Participant (Trial ~ Intercept)		0.28
Observations	8.56	

Model fit

	Marginal	Conditional
R^2	0.056	0.553

Note. P-values for fixed effects have been calculated using Satterthwaite's approximations. Confidence intervals have been calculated using the Wald method.
Model equation: expectation ~ trial * interference * ability + (1 + trial | subject)

Table S3: Correlations of model parameters (winning computational model M5)

	**LR neg**	**attribution weight**	**feedback weight**
**LR pos**	ρ = -0.4, p_{FDR} = .005*	ρ = -0.06, p_{FDR} = .629	ρ = 0.2, p_{FDR} = .216
**LR neg**		ρ = 0.07, p_{FDR} = .629	ρ = -0.25, p_{FDR} = .137
**attribution weight**			ρ = -0.07, p_{FDR} = .629

Note. ρ = Spearman's rank correlation coefficient. Benjamini-Hochberg false discovery rate correction (FDR) was applied to correct *p*-values for multiple testing. The attribution weight and feedback weight parameters are not significantly correlated with the learning rates or with each other.

Supplementary Notes

Supplementary Note 1: Participant instruction

The following study examines cognitive estimation ability. You will soon be presented with estimation questions in various categories. These estimation categories are comparable in terms of average difficulty, but individuals may differ (sometimes significantly) in their estimation performance. The goal is to answer these questions as accurately as possible.

In addition to answering the estimation tasks, another goal is to predict your own estimation performance as accurately as possible. After each trial, you will receive feedback on how well you performed on the task. In some trials, the computer may manipulate your feedback, meaning you might see feedback indicating a better or worse performance than you actually had. Your task in these trials is to determine whether the feedback reflects your actual performance or has been manipulated. In each trial, you will be informed whether it is possible that the computer has manipulated the feedback.

Here is a summary of the most important rules:

- There will be 4 estimation categories: heights, weights, distances, and quantities.
- In 2 of these categories, your feedback may be manipulated by the computer; in the other 2 categories, it will not.
- Always try to predict your actual performance as accurately as possible, even in trials where the feedback might be manipulated.
- On each trial, you will be explicitly informed whether the feedback could be manipulated by the computer.

Supplementary Note 2: Sample size determination and post-hoc sensitivity analysis

An a priori power analysis was conducted using G*Power version 3.1.9.7 (Faul et al. 2007) to determine the required sample size. We initially planned to analyze four learning rates (one learning rate per experimental condition) in a general linear model with the two factors Interference (agent vs. no agent) x Ability (high ability vs low ability) and depression scores as a covariate. This design was approximated using an F-test for fixed effects (ANOVA: fixed effects, special, main effects and interactions). Assuming a medium to large effect size ($f = 0.35$), an alpha level of .05, and desired power of .80, the analysis indicated a required total sample size of $N = 67$. This calculation focused on detecting the interaction between Interference and Ability, which was the primary effect of interest.

However, we deviated from the original analysis plan because the winning computational model included a different set of parameters, namely, two learning rates for positive and negative prediction errors across all experimental conditions, as well as an additional attribution weight factor.

Associations between learning bias parameters and individual differences (depression and self-esteem) were examined using Spearman rank correlations. A post hoc sensitivity analysis was conducted using G*Power, approximating the test using a bivariate correlation model. Assuming a two-tailed α level of .05 and power $(1 - \beta) = .80$, the available sample size ($N = 64$) allowed detection of correlations of at least $r = .34$. This indicates that the study was sufficiently powered to detect medium associations between individual differences in depression/self-esteem and learning biases, whereas smaller correlations may not have been reliably detected.

Associations between attributional bias and individual differences (depression and self-esteem) were now analyzed using a linear mixed-effects model. Because G*Power does not directly support mixed-effects models, the analysis was approximated using a linear multiple regression framework (R^2 increase), focusing on the unique contribution of the predictor of interest (e.g., depression or self-esteem). Assuming an alpha level of .05 and power $(1 - \beta) = .80$, the available sample size ($N = 64$) allowed detection of effects of at least $f^2 = 0.13$. This again corresponds to approximately $r = .34$, indicating that the study was powered to detect medium effects, whereas smaller effects may not have been reliably detected. This approximation does not account for the multilevel structure of the data and should therefore be interpreted cautiously. Moreover, given this sensitivity threshold, statistically significant effects close to this boundary may be subject to effect size inflation.

Supplementary References

Faul, Franz, Edgar Erdfelder, Albert-Georg Lang, and Axel Buchner. 2007. “G*Power 3: A Flexible Statistical Power Analysis Program for the Social, Behavioral, and Biomedical Sciences.” *Behavior Research Methods* 39 (2): 175–191.

Vehtari, Aki, Tommi Mononen, Ville Tolvanen, Tuomas Sivula, and Ole Winther. 2016. “Bayesian Leave-One-out Cross-Validation Approximations for Gaussian Latent Variable Models.” *Journal of Machine Learning Research: JMLR* 17 (103): 1–38.
